# Activation of spinal dorsal horn astrocytes by noxious stimuli involves descending noradrenergic signaling

**DOI:** 10.1186/s13041-021-00788-5

**Published:** 2021-05-10

**Authors:** Riku Kawanabe, Kohei Yoshihara, Izuho Hatada, Makoto Tsuda

**Affiliations:** 1 Department of Molecular and System Pharmacology, Graduate School of Pharmaceutical Sciences, Kyushu University, 3-1-1 Maidashi, Higashi-ku, Fukuoka, 812-8582 Japan; 2 Laboratory of Genome Science, Biosignal Genome Resource Center, Institute for Molecular and Cellular Regulation, Gunma University, 3-39-15 Showa-machi, Maebashi, Gunma 371-8512 Japan

## Abstract

Astrocytes are critical regulators of neuronal function in the central nervous system (CNS). We have previously shown that astrocytes in the spinal dorsal horn (SDH) have increased intracellular Ca^2+^ levels following intraplantar injection of the noxious irritant, formalin. However, the underlying mechanisms remain unknown. We investigated these mechanisms by focusing on the role of descending noradrenergic (NAergic) signaling because our recent study revealed the essential role of the astrocytic Ca^2+^ responses evoked by intraplantar capsaicin. Using in vivo SDH imaging, we found that the Ca^2+^ level increase in SDH astrocytes induced by intraplantar formalin injection was suppressed by ablation of SDH-projecting locus coeruleus (LC)-NAergic neurons. Furthermore, the formalin-induced Ca^2+^ response was dramatically decreased by the loss of α_1A_-adrenaline receptors (ARs) in astrocytes located in the superficial laminae of the SDH. Moreover, similar inhibition was observed in mice pretreated intrathecally with an α_1A_-AR-specific antagonist. Therefore, activation of α_1A_-ARs via descending LC-NAergic signals may be a common mechanism underlying astrocytic Ca^2+^ responses in the SDH evoked by noxious stimuli, including chemical irritants.

**Supplementary Information:**

The online version contains supplementary material available at 10.1186/s13041-021-00788-5.

Astrocytes, which are abundant glial cells in the CNS, have become increasingly recognized as critical elements regulating neuronal function [1] including somatosensory information processing in the spinal dorsal horn (SDH) [2, 3] and brain [4]. By using a method of in vivo Ca^2+^ imaging in the SDH [5], several studies have shown that SDH astrocytes have increased intracellular Ca^2+^ levels ([Ca^2+^]_i_) following strong mechanical pressure (pinch) to the hindpaw [6] and intraplantar injection of chemical irritants (capsaicin and formalin) [7, 8], suggesting that SDH astrocytes respond to noxious stimuli in the periphery. However, the mechanism underlying the increase in astrocytic [Ca^2+^]_i_ is not fully understood. Our recent study demonstrated that [Ca^2+^]_i_ increases in SDH astrocytes after intraplantar capsaicin are mediated by the activation of α_1A_-adrenaline receptors (α_1A_-ARs) through descending noradrenergic (NAergic) neurons from the locus coeruleus (LC) to the SDH [7]. However, whether the α_1A_-AR-mediated descending LC-NAergic signals commonly contribute to astrocytic Ca^2+^ responses evoked by noxious stimuli remains unclear. In this study, we investigated astrocytic Ca^2+^ responses to noxious irritant formalin using multiple approaches, including in vivo Ca^2+^ imaging, circuit-specific neuronal ablation, conditional gene knockout, and pharmacological intervention.

For in vivo Ca^2+^ imaging in SDH astrocytes, the Ca^2+^ indicator, GCaMP6m, was selectively expressed in SDH astrocytes following microinjection of an adeno-associated virus (AAV) vector expressing GCaMP6m under the control of the astrocytic promoter, gfaABC_1_D, into the left SDH (Additional file 1: Figure S1; Additional file 2), as reported previously [7, 8]. Using GCaMP6m-expressing mice under anesthesia, we confirmed that intraplantar injection of formalin, but not vehicle, induced robust increases in [Ca^2+^]_i_ in SDH astrocytes (Fig. 1a). To examine the involvement of the descending LC-NAergic pathway, we employed a circuit-specific ablation method using diphtheria toxin (DTX) and its receptor (DTR). To ablate SDH-projecting LC-NAergic neurons, AAVretro-Cre was microinjected into the left SDH of wild-type mice, and AAV-FLEX-DTR-EGFP or AAV-FLEX-AcGFP (control) was injected into the bilateral LC (Fig. 1b). In these mice, GFP expression was observed in the LC, and GFP^+^ LC neurons were immunolabeled with an antibody for tyrosine hydroxylase (TH), a marker for catecholaminergic neurons (mostly NAergic neurons in the LC) (Fig. 1c). Systemic administration of DTX eliminated GFP^+^ LC neurons in mice with AAV-FLEX-DTR-EGFP, but not in those with AAV-FLEX-AcGFP (Fig. 1c). In GCaMP6m-expressing mice with an ablation of descending LC-NAergic neurons, we found that the percentage of SDH astrocytes with increased [Ca^2+^]_i_ evoked by intraplantar formalin injection was significantly lower (Fig. 1d). The average trace of Ca^2+^ responses and the area under the curve (AUC) of Ca^2+^ traces from individual SDH astrocytes during the first 600 s after formalin injection were also suppressed. These results indicate that the descending LC-NAergic pathway contributes to formalin-induced astrocytic Ca^2+^ responses in SDH.

**Fig. 1:**
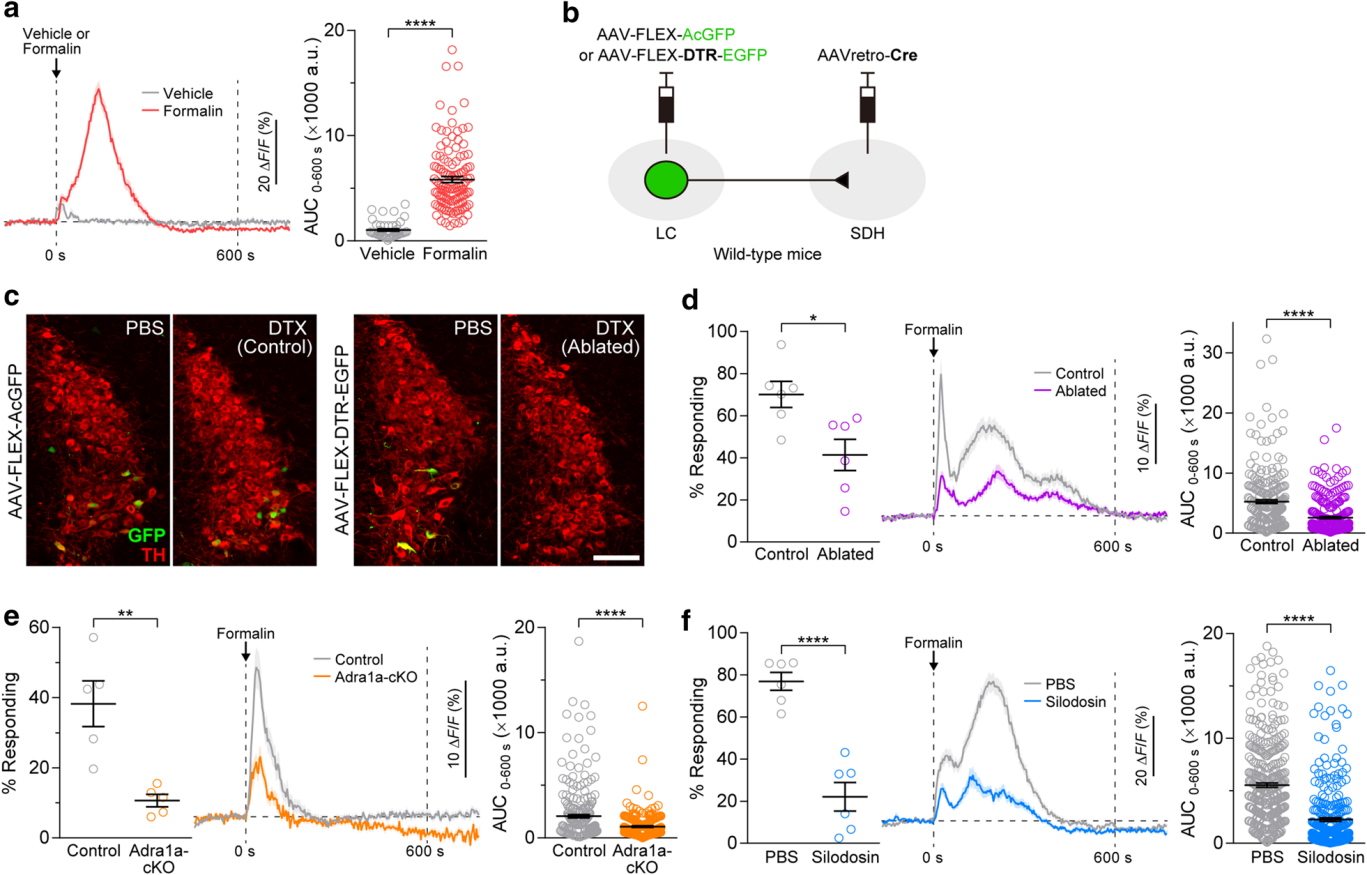
Intraplantar injection of formalin activates SDH astrocytes via α_1A_-ARs through descending LC-NAergic signals.** a** Averaged trace and AUC during the first 600 s (AUC_0–600 s_) of astrocytic Ca^2+^ signals in the SDH after intraplantar injection of vehicle or formalin (vehicle, *n* = 47 ROIs, 4 mice; formalin, *n* = 123 ROIs, 4 mice, ****P < 0.0001, Mann–Whitney U test). **b** Schematic illustration of retrograde transduction strategy in descending LC-NAergic neurons using the FLEX-switch system. **c** Representative images of LC-NAergic neurons in mice treated with PBS or DTX administration. GFP (green), and TH (red). **d**–**f** SDH astrocytic Ca^2+^ responses by formalin in mice with ablation of descending LC-NAergic neurons (**d**), conditional knockout of α_1A_-ARs in *Hes5*^+^ astrocytes (Adra1a-cKO; *Hes5-CreERT2*;*Adra1a*^flox/flox^) compared with control mice (control; *Adra1a*^flox/flox^) (**e**), and pretreatment intrathecally with PBS or silodosin (3 nmol) (**f**). Percentage of responding astrocytes (**d** control, *n* = 6 mice; ablated, *n* = 6 mice; **e**: control, *n* = 5 mice; Adra1a-cKO, *n* = 5 mice; **f** PBS, *n* = 6 mice; silodosin, *n* = 6 mice, *P < 0.05, **P < 0.01, ****P < 0.0001, unpaired *t*-test); averaged trace and AUC (**d** control, *n* = 255 ROIs; ablated, *n* = 263 ROIs; **e** control, *n* = 253 ROIs; Adra1a-cKO, *n* = 224 ROIs; **f** PBS, *n* = 364 ROIs; silodosin, *n* = 296 ROIs, ****P < 0.0001, Mann–Whitney U test). Data show the mean ± SEM

We previously identified α_1A_-AR as an astrocyte-expressing receptor necessary for Ca^2+^ responses evoked by intraplantar capsaicin [7]. Consistent with our previous study [7], immunohistochemical analysis confirmed that 96.5 ± 1.9% of SDH astrocytes expressed α_1A_-ARs (Additional file 1: Figure S2). Thus, we examined the role of α_1A_-AR using *Hes5-CreERT2*;*Adra1a*^flox/flox^ mice (treated with tamoxifen) that lack this receptor in SDH astrocytes, especially localized in superficial laminae [7]. The number of SDH astrocytes with increased [Ca^2+^]_i_ by formalin in *Adra1a*^flox/flox^ control mice (Control) was dramatically decreased in *Hes5-CreERT2*;*Adra1a*^flox/flox^ mice (Adra1a-cKO) (Fig. 1e). The average trace and AUC for Ca^2+^ responses were also lower in Adra1a-cKO mice than in control mice. Adra1a-cKO mice treated with tamoxifen also lack α_1A_-AR expression in brain *Hes5*^+^ astrocytes [7]. To determine the importance of α_1A_-ARs in the spinal cord, we intrathecally administered the α_1A_-AR-specific antagonist, silodosin, before formalin injection. Silodosin-pretreated mice also showed marked inhibition of the formalin-induced astrocytic Ca^2+^ responses (the percentage of SDH astrocytes with [Ca^2+^]_i_ increases, the average trace of Ca^2+^ responses, and their AUCs) (Fig. 1f). Taken together, the Ca^2+^ responses in SDH astrocytes following formalin injection are mediated by the activation of α_1A_-ARs through descending LC-NAergic signals.

In this study, we demonstrate for the first time that intraplantar injection of the noxious irritant, formalin, activates SDH astrocytes (especially the *Hes5*^+^ subset) via α_1A_-ARs stimulated by descending LC-NAergic signaling. Previous data showing induction of the neuronal activity marker c-FOS in LC-NAergic neurons [9] supports our findings. Given that astrocytic Ca^2+^ responses in the SDH after intraplantar capsaicin are mediated by α_1A_-AR-mediated descending LC-NAergic signaling [7], this raises the possibility that this signaling pathway from the LC-NAergic neurons to SDH astrocytes is a common mechanism for astrocytic Ca^2+^ responses in the SDH evoked by noxious chemical irritants. However, the decrease in the number of responding astrocytes was slightly lower in mice with LC-NAergic neuron ablation than in mice with conditional α_1A_-AR-knockout and silodosin pretreatment. This could be due to incomplete ablation of LC-NAergic neurons projecting to the 4th lumbar SDH where astrocytic Ca^2+^ responses were monitored or the involvement of other descending NAergic pathways, for example, from regions A5 and A7 (although the LC is the main source of NA in the SDH [10]). In addition, considering the residual astrocytic Ca^2+^ responses observed in mice either with genetic knockout or pharmacological blockade of α_1A_-ARs, it seems that other neurotransmitters, such as glutamate, GABA, and ATP, which are known to cause astrocytic Ca^2+^ elevations [11], may also be involved. Nevertheless, our findings indicate that α_1A_-AR-mediated descending LC-NAergic signals are a primary driver of Ca^2+^ responses in SDH astrocytes evoked by noxious stimuli.

In this study, there were different patterns of the average traces of Ca^2+^ responses after intraplantar formalin injection among experiments. The reason for this difference remains unclear. Nevertheless, Ca^2+^ responses during several minutes after the injection are commonly observed and are consistent with our previous data [8]. However, Ca^2+^ responses in *Adra1a*^flox/flox^ and *Hes5-CreERT2*;*Adra1a*^flox/flox^ mice were different from others. It may involve a genetic factor (and/or tamoxifen treatment) because the genetic background of *Adra1a*^flox/flox^ mice was derived from BDF1 [(C57BL/6 × DBA/2)F1] strain and these mice were not fully backcrossed on the C57BL/6 background, while other experiments used C57BL/6 mice.

Formalin is used as a model for acute and persistent inflammatory pain associated with peripheral tissue injury. The role of spinal NAergic signals in formalin-induced pain has been examined in many studies [12, 13], but it remains controversial. For example, intrathecal treatment with α_2_-AR agonists reduces formalin pain [12, 14], intrathecal treatment with anti-dopamine-β-hydroxylase antibody-conjugated saporin, which kills SDH-projecting NAergic neurons, attenuates formalin pain [15]. An explanation for this discrepancy may be partly associated with the action of NA in SDH astrocytes. It should be noted that we measured the astrocytic Ca^2+^ responses for the first 10 min after formalin injection, a time period that corresponds to acute phase of formalin-induced nociceptive behavior. Further investigations using a tool to manipulate Ca^2+^ responses specifically in *Hes5*^+^ SDH astrocytes will uncover their in vivo role in nociceptive information processing and behaviors evoked by formalin.

## Supplementary Information

**Additional file 1: Figure S1**. Immunohistochemical identification of GCaMP6m-expressing cells in the SDH. Spinal cord sections from mice with microinjection of AAV-gfaABC_1_D-GCaMP6m into the SDH were immunostained by cell-type-specific markers (SOX9 and GFAP, astrocytes; NeuN, neurons; IBA1, microglia; APC, oligodendrocytes) (red). Note that GCaMP6m-expressing cells (green) were positive to astrocyte markers (SOX9 and GFAP) but were negative to other markers (NeuN, IBA1 and APC). Scale bar, 20 μm. **Figure S2.** Immunohistochemical analysis of α_1A_-AR expression in SDH astrocytes. Immunofluorescence of α_1A_-AR (green) and GFAP (magenta) in the SDH of wild-type mice. Scale bar, 20 μm. Percentage of α_1A_-AR^+^ astrocytes per total SDH astrocytes (*n* = 166 cells, 9 slices from 3 mice). Data show the mean ± SEM.

**Additional file 2**. Methods.

## Data Availability

All data generated or analyzed during this study are included in this published article and its Additional file.

## Abbreviations

AAV: Adeno-associated virus
AR: Adrenaline receptor
AUC: Area under the curve
DTR: Diphtheria toxin receptor
DTX: Diphtheria toxin
Hes5: Hairy and enhancer of split 5
LC: Locus coeruleus
NA: Noradrenaline
SDH: Spinal dorsal horn
TH: Tyrosine hydroxylase
