# Supplementary material for: Activation of spinal dorsal horn astrocytes by noxious stimuli involves descending noradrenergic signaling
Source: Mol Brain. 2021 May 10;14:79. doi: 10.1186/s13041-021-00788-5 (PMC8108464; doi:10.1186/s13041-021-00788-5)
Supplement: Supplementary file 1 — Additional file 1: Figure S1. Immunohistochemical identification of GCaMP6m-expressing cells in the SDH. Spinal cord sections from mice with microinjection of AAV-gfaABC1D-GCaMP6m into the SDH were immunostained by cell-type-specific markers (SOX9 and GFAP, astrocytes; NeuN, neurons; IBA1, microglia; APC, oligodendrocytes) (red). Note that GCaMP6m-expressing cells (green) were positive to astrocyte markers (SOX9 and GFAP) but were negative to other markers (NeuN, IBA1 and APC). Scale bar, 20 μm. Figure S2. Immunohistochemical analysis of α1A-AR expression in SDH astrocytes. Immunofluorescence of α1A-AR (green) and GFAP (magenta) in the SDH of wild-type mice. Scale bar, 20 μm. Percentage of α1A-AR+ astrocytes per total SDH astrocytes (n = 166 cells, 9 slices from 3 mice). Data show the mean ± SEM. [file 13041_2021_788_MOESM1_ESM.docx]

**Additional file 1**

**Supplementary Figure 1**


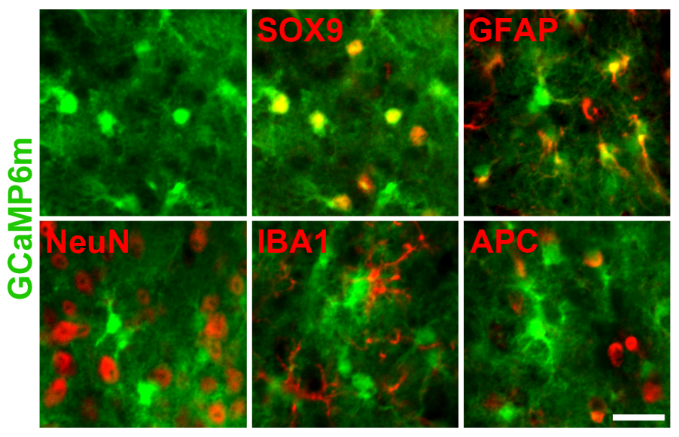


**Immunohistochemical identification of GCaMP6m-expressing cells in the SDH.**

Spinal cord sections from mice with microinjection of AAV-gfaABC_1_D-GCaMP6m into the SDH were immunostained by cell-type-specific markers (SOX9 and GFAP, astrocytes; NeuN, neurons; IBA1, microglia; APC, oligodendrocytes) (red). Note that GCaMP6m-expressing cells (green) were positive to astrocyte markers (SOX9 and GFAP) but were negative to other markers (NeuN, IBA1 and APC). Scale bar, 20 μm.

**Supplementary Figure 2**


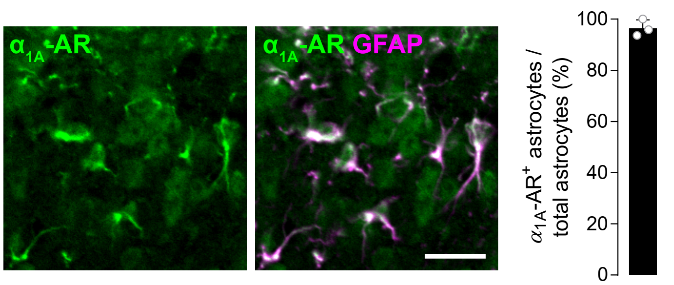


**Immunohistochemical analysis of α_1A_-AR expression in SDH astrocytes.**

Immunofluorescence of α_1A_-AR (green) and GFAP (magenta) in the SDH of wild-type mice. Scale bar, 20 μm. Percentage of α_1A_-AR^+^ astrocytes per total SDH astrocytes (*n* = 166 cells, 9 slices from 3 mice). Data show the mean ± SEM.
