# Supplementary material for: Activation of spinal dorsal horn astrocytes by noxious stimuli involves descending noradrenergic signaling
Source: Mol Brain. 2021 May 10;14:79. doi: 10.1186/s13041-021-00788-5 (PMC8108464; doi:10.1186/s13041-021-00788-5)
Supplement: Supplementary file 2 — Additional file 2. Methods. [file 13041_2021_788_MOESM2_ESM.docx]

**Additional file 2**

**Methods**

**Animals**

Male C57BL/6J mice (CLEA Japan) and *Adra1a*^flox/flox^ mice [1][1] were used. The genetic background of *Adra1a*^flox/flox^ mice was derived from BDF1 [(C57BL/6 × DBA/2)F1] strain, and in this study we used *Adra1a*^flox/flox^ mice that were backcrossed one time onto the C57BL/6 background. *Hes5-CreERT2* mice [2] were provided by Prof. Verdon Taylor (University of Basel). For induction of Cre recombinase, *Hes5-CreERT2* mice (8–12 weeks old) were given an i.p. injection of 2 mg tamoxifen (Sigma) dissolved in 100 μL corn oil (Wako) once a day for 10 days [1]. We used tamoxifen-injected mice for further analysis at least 7 days after the last injection. All mice used were 8–12 weeks old at the start of each experiment and were housed at 22 ± 1 °C with a 12-h light–dark cycle with food and water ad libitum. All animal experiments were conducted according to relevant national and international guidelines contained in the ‘Act on Welfare and Management of Animals’ (Ministry of Environment of Japan) and ‘Regulation of Laboratory Animals’ (Kyushu University) and under the protocols approved by the Institutional Animal Care and Use committee review panels at Kyushu University.

**Recombinant adeno-associated virus (rAAV) production**

The AAV vectors were produced according to a previous method [1, 3]. The gene encoding GCaMP6m (amplified from Addgene #40754) [4] was subcloned into the pENTR plasmid (Thermo Fisher Scientific). We cloned AcGFP, EGFP and diphtheria toxin receptor (DTR) (kindly provided by Prof. Kenji Kohno, Nara Institute of Science and Technology) [5]. To produce the AAV vector for the Cre-switch system [6], vectors containing the promoters encoding EF1α was generated from pAAV-CA-FLEX (Addgene #38042) by substituting the promoter. We then inserted AcGFP, EGFP and DTR into pAAV-EF1α-FLEX. Furthermore, each AAV expression plasmid pZac2.1 (Penn Vector Core) carrying GCaMP6m driven by the gfaABC_1_D promoter was constructed to target astrocytes, and pZac2.1 carrying the Cre recombinase driven by enhanced synapsin promoter (ESYN) was constructed to target neurons. The rAAV2-retro helper plasmid was purchased from Addgene (#81070) [7].

**Intra-SDH and intra-LC injection of AAV vectors**

The used AAV vectors and their titers were as follows: AAV2/9-gfaABC_1_D-GCaMP6m, 8.5 × 10^12^ genome copies (GC)/mL; AAV2/retro-ESYN-Cre, 3.0 × 10^12^ GC/mL; AAV2/9-EF1α-FLEX-AcGFP and AAV2/9-EF1α-FLEX-DTR-EGFP, 2.0 × 10^12^ GC/mL. According to our previously described method [1, 3, 8], rAAV solutions were microinjected into the left side of the SDH (200−250 μm in depth from the surface of the dorsal root entry zone) using a Micro4 Micro Syringe Pump Controller (World Precision Instrument) (approximately 500 nL). For intra-LC injection, AAV vector solutions were bilaterally injected (approximately 300 nL per the unilateral site) in the LC [anteroposterior (AP), 5.6 mm; mediolateral (ML), ± 1 mm; dorsoventral (DV) 3.7 mm], as previously reported [1]. Virus-injected mice were used for experiments at least 21 days after the last AAV vector injection.

**Immunohistochemistry**

Mice were deeply anesthetized with an i.p. injection of pentobarbital and transcardially perfused with PBS followed by ice-cold 4% paraformaldehyde (PFA)/PBS. The transverse L4 segments of the spinal cord and brainstem were removed, postfixed in the same fixative for 3 h (spinal cord) or 24 h (brainstem) at 4 °C, and placed in 30% sucrose solution for 48 h at 4 °C. Sections of transverse L4 spinal cord and coronal brain (30 μm) were stained according to our previous method [1]. Primary and secondary antibodies used were listed below. Primary antibodies: monoclonal rat anti-GFAP (1:2000, Invitrogen, 13-0300), polyclonal goat anti-SOX9 (1:2000, R&D Systems, AF3075), monoclonal mouse anti-NeuN-Alexa Fluor 555 conjugate (1:2000, Millipore, MAB377A5), polyclonal rabbit anti-IBA1 (1:4000, Wako, 019-19741), monoclonal mouse anti-APC (1:500, Calbiochem, OP80), polyclonal rabbit anti-GFP (1:2000, MBL, 598), polyclonal sheep anti-TH (1:1000, Millipore, AB1542), polyclonal rabbit anti-α_1A_-AR (1:200, Alomone labs, AAR-015) and secondary antibodies: Alexa Fluor 546 (1:1000, Molecular Probes, A11030, A11035, A11056, A11081, A21098). Immunofluorescence images were obtained with a confocal laser microscope (LSM700, Carl Zeiss).

**Drug administration**

For intraplantar injection of formalin, a 30-G needle attached to a Hamilton microsyringe was inserted to the plantar surface of the left hindpaw. For intrathecal injection of silodosin, a 30-G needle attached to a Hamilton microsyringe was inserted between the L5/L6 vertebrae and then punctured through the dura [9]. Silodosin (3 nmol in 5 μL PBS, Wako) was intrathecally injected 30–40 min before *in vivo* Ca^2+^ imaging [1]. For ablation of descending LC-NAergic neurons, 3 weeks after intra-LC injection of AAV vectors, DTX (10 μg/kg, Sigma) was dissolved in saline and was administered i.p. once a day for 2 days.

***In vivo* Ca^2+^ imaging with two-photon microscopy**

We used a previously described method for chamber implantation [1, 8]. Under isoflurane anesthesia, two-photon Ca^2+^ imaging was performed using an Olympus FV1000 with a ×25/1.05 numerical aperture water-immersion lens (Olympus) and a MaiTaiHP Ti:sapphire laser (Spectra Physics) tuned at 900 nm for two-photon excitation of GCaMP6m. The laser power was set to the lowest level (~20 mW), and images were acquired with a frequency of 0.30−0.33 Hz to avoid phototoxicity. During two-photon imaging, mice were placed on a heating pad (37 °C). Ca^2+^ imaging was performed at a depth of ~50 μm in the left lumbar SDH. Mice were injected intraplantar with 20 μL of 5% formalin (Wako) dissolved in saline. *In vivo* imaging of one mouse from each experimental group was performed in the same day, and formalin-induced SDH astrocytic Ca^2+^ responses were recorded 30−40 min.

For data processing and analysis [1, 8], videos were imported into ImageJ (http://rsbweb.nih.gov/ij/), and motion artifacts were corrected using TurboReg [10]. The size of the region of interest (ROI) was set to 10 µm in diameter. The change in fluorescence was expressed as a relative percentage change, Δ*F*/*F* = 100 × (*F_t_* − *F*_0_)/*F*_0_, where *F_t_* is the fluorescence at time *t* and *F*_0_ is the baseline average for 90 s before formalin injection. ROIs were manually selected based on cell morphology and on ≥ 30% Δ*F*/*F* Ca^2+^ transients at least once during the entire imaging session or just after euthanasia with an overdose of pentobarbital (almost all astrocytes elicit Ca^2+^ transients immediately after euthanasia). ROI analysis was performed using the multi measure plugin in ImageJ. Calculating the AUC was performed using Prism7 (GraphPad).

**Statistical analysis**

Statistical analyses were performed using Prism 7 (GraphPad). All data are shown as the mean ± SEM. Statistical significance of differences was determined using two-tailed unpaired *t*-test or Mann-Whitney U test. Differences were considered significant at *P* < 0.05.

**References**

1. Kohro Y, Matsuda T, Yoshihara K, Kohno K, Koga K, Katsuragi R, et al. Spinal astrocytes in superficial laminae gate brainstem descending control of mechanosensory hypersensitivity. Nat Neurosci. 2020;23(11):1376-87.

2. Lugert S, Vogt M, Tchorz JS, Müller M, Giachino C, Taylor V. Homeostatic neurogenesis in the adult hippocampus does not involve amplification of Ascl1(high) intermediate progenitors. Nat Commun. 2012;3:670.

3. Kohro Y, Sakaguchi E, Tashima R, Tozaki-Saitoh H, Okano H, Inoue K, et al. A new minimally-invasive method for microinjection into the mouse spinal dorsal horn. Sci Rep. 2015;5:14306.

4. Chen TW, Wardill TJ, Sun Y, Pulver SR, Renninger SL, Baohan A, et al. Ultrasensitive fluorescent proteins for imaging neuronal activity. Nature. 2013;499(7458):295-300.

5. Saito M, Iwawaki T, Taya C, Yonekawa H, Noda M, Inui Y, et al. Diphtheria toxin receptor-mediated conditional and targeted cell ablation in transgenic mice. Nat Biotechnol. 2001;19(8):746-50.

6. Watabe-Uchida M, Zhu L, Ogawa SK, Vamanrao A, Uchida N. Whole-brain mapping of direct inputs to midbrain dopamine neurons. Neuron. 2012;74(5):858-73.

7. Tervo DG, Hwang BY, Viswanathan S, Gaj T, Lavzin M, Ritola KD, et al. A Designer AAV Variant Permits Efficient Retrograde Access to Projection Neurons. Neuron. 2016;92(2):372-82.

8. Yoshihara K, Matsuda T, Kohro Y, Tozaki-Saitoh H, Inoue K, Tsuda M. Astrocytic Ca^2+^ responses in the spinal dorsal horn by noxious stimuli to the skin. J Pharmacol Sci. 2018;137(1):101-4.

9. Hylden JL, Wilcox GL. Intrathecal morphine in mice: a new technique. Eur J Pharmacol. 1980;67(2-3):313-6.

10. Thévenaz P, Ruttimann UE, Unser M. A pyramid approach to subpixel registration based on intensity. IEEE Trans Image Process. 1998;7(1):27-41.
